# Accidental Loss of Drainage-tubes in Pleural Cavity.—Removal by Operation

**Published:** 1877-04

**Authors:** John H. Ripley


### MISCELLANEOUS.

### St. Francis' Hospital---Accidental Loss of Drainage-Tubes in Pleural Cavity---Successful Removal by Operation.

Service of Dr. John H. Ripley. (Reported by Charlse F. Stillman, M. D., Assistant House Physician.)

Edward Dorsey, 21, single, Ireland, cotton-spinner, was admitted January 11th, with the following history: A year ago last November was attacked with pleurisy of the left side, which was treated without avail by counter-irritants, and in the following

April ʒxxxi. of clear serum were removed by aspiration. After the lapse of six weeks the effusion was found to be purulent, and an incision was made at the junction of the axillary line and seventh intercostal space, ℥xxvi. of creamy pus being evacuated, and the wound allowed to heal. In July the incision was renewed and nearly ʒl. removed ; a drainage-tube inserted, slipped into the patient's pleural cavity the ensuing night. The wound has been draining steadily ever since, but last Christmas he was so unfortunate as to lose another tube in a similar manner, and he entered this hospital January 11th, for relief, his attending surgeon having previously attempted their removal without success. On admission, the patient is well nourished, and complains only of occasional pain in the seventh intercostal space, radiating towards the sternum.

The left side of the chest measures two and a half inches less than the right in the xiphoid line, and possesses greatly diminished lateral expansion, with partial effacement of the intercostal spaces by approximation of the ribs. Vocal fremitus on the affected side increased in the mammary, and absent in the infraclavicular and axillary regions. Extra resonance on percussion in the infraclavicular region, while in the axillary region there is dulness, which inferiorly becomes flatness, the mammary and infra-mammary regions being slightly extra-resonant.

*Auscultation* reveals very feeble respiration *anteriorly*, with absence of the respiratory murmur over the lower fourth, together with an occasional sticky friction sound. *Posteriorly* in the upper half the respiration is prolonged, with absence of respiratory sound over the lower half and metalic tinkle.

January 18th.—It was decided to operate for the removal of the tubes, and accordingly an incision was made by Dr. Ripley in the seventh intercostal space, beginning at a point six and a half inches from the medial line and extending posteriorly a distance of four inches. The seventh and eighth ribs were so closely approximated that, at the point where the drainage-tube had been in constant contact with them, a portion of each had necrosed, and it was found necessary to excise a portion of the eighth rib before the proper instruments could be introduced into the pleural cavity. An angular fracture was also found on the seventh rib superiorly, possibly caused by an attempt to force the ribs apart in the operation he sustained before admission. The pleural cavity having been entered after the removal of the dead fragments of bone, its interior was carefully explored by means of uterine forceps, and an Emmet's silver probe so bent at the extremity as to form a hook, and yet so flexible as to offer but little danger of perforating the lung.

After repeated and cautious attempts, the two tubes were found about an inch apart, lying posterior and superior to the incision. The first was found and removed by the forceps, the second by the probe; both being constructed of the material ordinarily used for gum-elastic catheters. The tube retained in the chest since last July measured seven inches in length, was about the diameter of a large goose-quill, and somewhat blackened, although but very slightly absorbed, by the fluids in the chest. The other tube measured six inches in length and appeared very bright and fresh. The numerous adhesions and thick layers of fibrine contained in the sack rendered the operation quite difficult. The patient was placed in bed and $\frac{1}{4}$ gr. morphine injected hypodermically every two hours until midnight, after which he slumbered easily until morning. The next day the temperature did not obtain more than 100° F., and the highest point reached the second day was 100 1-5° F. in the rectum, since which time it has not exceeded 100°. On the 23d, a drainage-tube was inserted in the lower portion of the incision, the upper having united by first intention, and the patient has since been doing well, the amount of discharge daily diminishing.—*Med. Record.*

